# Presentation of Study Results: Kalkbrenner et al. Respond

**DOI:** 10.1289/ehp.1205556R

**Published:** 2012-08-31

**Authors:** Amy E. Kalkbrenner, Joe M. Braun, Maureen S. Durkin, Matthew J. Maenner, Christopher Cunniff, Julie L. Daniels

**Affiliations:** 1 Zilber School of Public Health, University of Wisconsin–Milwaukee, Milwaukee, Wisconsin, E-mail: kalkbren@uwm.edu; 2 Department of Environmental Health, Harvard School of Public Health, Boston, Massachusetts; 3 Department of Population Health Sciences, University of Wisconsin–Madison, Madison, Wisconsin; 4 Waisman Center, University of Wisconsin–Madison, Madison, Wisconsin; 5 Department of Pediatrics, University of Arizona College of Medicine, Tucson, Arizona; 6 Department of Epidemiology, University of North Carolina–Chapel Hill, Chapel Hill, North Carolina

We are grateful for the opportunity to comment on the importance of careful interpretation and communication of epidemiological results. We concur with Burstyn et al. that scientists have a great responsibility to present their findings accurately. Indeed, scientists have a duty not only to generate research results but also to engage in dialog about the interpretation and meaning of these results.

We also agree that our study on maternal smoking in pregnancy and the risk of autism spectrum disorders (ASDs) had limitations, including heterogeneous outcome groupings, residual confounding, and exposure misclassification (Kalkbrenner et al. 2012). We do not, however, share the conviction that these limitations void the validity and contribution of our study or that our results should not be accompanied with an interpretation.

Our study improved upon previous studies of tobacco exposures and ASDs in several ways. It incorporated population-based data from the United States. Much of the existing body of research on this subject has been conducted in Europe where social patterns of both smoking and ASD diagnoses differ from patterns in the United States. Furthermore, adjusting for maternal education, marital status, and maternal race/ethnicity was an improvement over many previous studies that were not able to adjust for these factors. Although residual social class confounding is possible, it would more likely have masked—rather than produced—the observed elevated associations. Finally, we included a large number of children with ASD and had some phenotypic information beyond a binary ASD/non-ASD classification. By including and reporting on several ASD functional subclassifications, we performed replicates within one study, increased the transparency and completeness of reporting, and enhanced the comparability with previous reports. The consistent pattern of results across subclassifications strengthened our interpretation that the overall pattern we observed was not due wholly to biases or random error.

Most puzzling to us is the null hypothesis testing approach described by Burstyn et al. in their letter, in which associations were dichotomized to conclude a “link” when confidence limits excluded the null value, or “no link” otherwise. This dichotomy of complex results is not only a grave oversimplification, but it is awash with assumptions that do not hold in observational epidemiology (Poole 2001; Savitz 1993).

**Table 1:** Meta-analytic perspective showing adjusted prevalence ratios (95% confidence limits).

ASD subclassification	Kalkbrenner et al.	Lee et al.	Combined^a^
ASD without co-occuring intellectual disabilities	1.14 (0.08, 1.47)	1.13 (0.95, 1.25)	1.13 (1.03, 1.24)
ASD with co-occuring intellectual disabilities	0.72 (0.53, 0.98)	0.91 (0.78, 1.06)	0.87 (0.76, 1.00)
^a^Data were combined using the EpiSheet tool (Rothman 2011) following the method of Fleiss (1993).										

Instead, we prefer a more meta-analytic mindset, in which a given study is considered as a contribution to a broader literature, with the weight of contribution proportional to its precision. Valid and precise results are interpreted for their public health or clinical importance, judged by the magnitude of effect.

As an illustration of the meta-analytic perspective, with no intention of definitively answering whether maternal smoking causes any subgroup of ASDs, we have performed a simple combination of results from our study (Kalkbrenner et al. 2012) with those from a similar recent population-based study of maternal smoking in pregnancy and ASDs (Lee et al. 2011). Using strict null-hypothesis testing interpretation, all original adjusted prevalence ratios support “no link” because the 95% confidence limits include the null value of 1.0 (Table 1). In contrast, a more nuanced interpretation of results, focusing on patterns of magnitude and precision, yields a conclusion that associations between maternal smoking and ASD may differ by the presence of co-occurring intellectual disabilities, consistent with the meta-analytic combined results.

In summary, the quality of our data, analytic approach, and interpretation improved upon previous studies of this important question. It is always possible to go further, and we are intrigued by the suggestion of Burstyn et al. to conduct an extended, multiple-bias sensitivity analysis. We explored the impact of underrecognition of ASD varying by social class (an important bias not previously addressed in the literature), but we did not evaluate this error together with the underreporting of tobacco use in pregnancy and residual social class confounding. The mathematical methods to explore and correct such interwoven biases are being developed and made available, opening up the possibility of better modeling these errors. This analysis should be explored in future work, and on this point perhaps we are in agreement with Burstyn et al.
